# Research engagement among black men with prostate cancer

**DOI:** 10.3332/ecancer.2016.695

**Published:** 2016-11-24

**Authors:** Charlotte Toms, Fidelma Cahill, Gincy George, Mieke Van Hemelrijck

**Affiliations:** King’s College London, Division of Cancer Studies, Cancer Epidemiology Group, London SE1 9RT, UK

**Keywords:** prostate cancer, black ethnicity, consent

## Abstract

**Background:**

Black men are three times more likely to develop prostate cancer (PCa) and often present with more aggressive disease. Nevertheless, black men are consistently underrepresented in research studies. We aimed to get more insight into the reasons for this reduced recruitment, as it is important for future research to include results that are also applicable to black men with PCa.

**Methods:**

Two focus groups (*n* = 10 and *n* = 6) of black males currently under treatment for PCa at Guys Hospital, London, UK were held to gather information regarding the understanding of and exposure to research, as well as the barriers and facilitators for recruitment into research studies.

**Results:**

Barriers to recruitment included; mistrust of researchers, lack of understanding of the research process and the mechanisms of PCa and a reliance on herbal medicine. Suggested facilitators for recruitment improvement included thorough explanations of the research process, media advertisement and word of mouth. Financial incentives were also discussed but received mixed reception.

**Conclusion:**

We uncovered a number of barriers to recruitment of black men with PCa into research and accompanying strategies for improving involvement. Many are consistent with the literature, emphasising that current efforts have not been successful in ameliorating the concerns of the black community. Beliefs in herbal medicine and aversion to financial incentives appear to be novel themes, and so further insight into these issues could prove beneficial.

## Introduction

Prostate cancer is the most common male cancer to affect men in the United Kingdom [1] – with black males having a threefold increased risk as compared to white men (166 and 56.4 per 100,000, respectively) [2]. It is not clear what explains these considerable differences, although it is likely to be a combination of genetic variations, lifestyle differences and medical care [3].

Despite this higher risk of prostate cancer, black minorities are consistently underrepresented in clinical trials [4]. This raises concerns regarding the benefits of current research to black males. A number of barriers to the recruitment of black males to research have been raised. A key theme is that of distrust in researchers. For instance, data from focus group interviews by Corbie-Smith et al. found lack of trust to be a major deterrent to participation in research, as individuals felt that researchers failed to consider the welfare of the participants [5]. There have been suggestions that these attitudes stem from historical studies, such as the Tuskegee Syphilis Study, in which researchers deliberately withheld treatment for Syphilis in the interest of studying disease progression [5]. Corbie-Smith et al. reported repeated references to this study throughout the focus group interviews [5]. Other barriers which are commonly suggested in the literature include lack of transportation, lack of cultural sensitivity in both recruitment approaches and the experimental procedures, lack of understanding of clinical trials and insufficient incentive availability [4].

This qualitative study was undertaken to further explore the reasons for low participation in research by black males with prostate cancer and to try to identify potential methods for facilitating research engagement.

## Materials and methods

### Participants and setting

Black prostate cancer patients currently under treatment at Guy’s Hospital (London, UK) were recruited by telephone and invited to participate in a focus group. Flyers advertising the focus groups were also displayed around the hospital. Thus, the list of patients invited consisted of black Afro-Caribbean patients currently attending prostate cancer clinics and black Afro-Caribbean patients who had seen posters advertising these focus groups in the urology and oncology clinics. Some individuals had previously taken part in research studies, but this was not a factor in deciding who would be invited to participate. In total, two focus group interviews were conducted between November 2015 and February 2016 (*n* = 10 and *n* = 6, respectively). The concept of the focus group was explained to the individuals upon invitation, and again at the beginning of the focus groups. Participants were also made aware of how the data would be utilised. All participants also completed a demographic questionnaire that obtained information on age, ethnicity, employment status, marital status and distance from the hospital. Participants were made aware how this data would be used and that it would remain anonymous prior to completion of the questionnaire. The focus groups were conducted by a research nurse and a research assistant. The focus groups covered previous exposure of the participants to research and the advertisement and understanding of research. In addition, there was exploration of participants’ attitudes towards taking part in research, including what would encourage or defer individuals from taking part. Focus group participants were asked a series of set questions (Table 1), but discussions between participants and, between participants and focus group leaders were unrestricted.

### Data analysis

The interviews were audio-taped and were transcribed verbatim. Transcripts were then analysed thematically [6]. This process involved the thorough examination of transcripts for features of interest or codes. The coded data were then collated into a series of broader themes, which were refined to ensure they were representative of the data.

### Literature search

To place the findings from the interviews in the wider context of the current literature, an additional literature search was undertaken. The search strategy involved a key word search of the Medline database (1966 to 2016): ‘African–Americans’, ‘African continental ancestry group’, ‘male’, ‘research’. No further exclusion criteria were applied.

## Results

Sixteen participants took part in the two focus groups. The demographics of the participants in the focus groups can be viewed in Table 2.

Upon being questioned about understanding of medical research, the two focus groups had differing views. Many of the individuals within the first focus group were confused regarding what was actually considered research. However, the general understanding of research within the second focus group was good. Similar separations of views occurred when participants were questioned regarding their participation in research. The majority of individuals within the first focus group confirmed that they had not been approached for research before. However, the majority of those in the second focus group had been approached.

## Factors preventing engagement in research

A number of factors were identified by the groups as having a negative impact on the decision to participate in research. Quotes from participants supporting these views are shown in Table 3.

### Mistrust

This was the major theme highlighted. While many of the participants did not express these concerns themselves, they were very aware of these opinions within the black community.

‘But it’s also to do with, people like us, you know, people of colour here, um, then there is a stigma also there. Thinking, you know, why are they trying to, you know, get us to do this and not go and ask somebody else. There’s that type of feeling, you know, because there’s a mistrust.’

Individuals expressed the wish to have the research fully explained; hence highlighting that having a lack of understanding regarding the research was undesirable. They wanted to know what was involved, and not feel as if information was being withheld.

### Lack of understanding

Some of the men were unsure about what constituted medical research. There was a little misunderstanding regarding the causes of prostate cancer and its underlying pathology and aetiology. For instance, one gentleman had strong views regarding the different Westernised environment of the United Kingdom, as compared to his country of birth, in the development of his prostate cancer. *‘We live, you see, I was born in Jamaica. Jamaica is in me. That’s where my roots and everything are. As soon as you come out of that and enter into a different environment, the climate is different, the food is different, the air is different, the structure. So our body becomes changed.’* Even when current evidence regarding environment and prostate cancer was explained to the patient, he did not appear to change his perceptions. These misunderstandings may need to be addressed in order to further appeal to individuals about the importance of research.

### Tendencies to favour herbal medicine

There were also considerable discussions regarding the use of herbal medicines. One gentleman expressed a clear interest in the use of herbal medication and confirmed his use of herbal medication in addition to the clinical trial drugs he was taking. *‘Well I’m looking at, because basically, we you see, in the West Indies, have lots of herbs and things like that what we boil when we have colds, when we’re sick in our stomach and all those kind of things...Take Aloe Vera for one. Take pineapple for one. Take Chocho, Ladyfingers and Moringa and Guinea Hen weed. And all these things.’*

## Factors encouraging engagement in research

A number of factors that would encourage engagement in research were also elicited. Quotes from participants regarding these themes can be viewed in Table 4.

### Comprehensive explanations of what the research entails

This was a key theme that was mentioned repeatedly by a number of participants. It seemed that a thorough explanation regarding what the research involves, and the procedures, was required in order to alleviate the mistrust between the participant and researcher.

‘To me it’s just about explaining really. I think. I only speak on my experience, I don’t know what everybody thinks. But um, but I think if things are fully explained. If I understand the benefits of the research, including statistics about you know, how many people are affected, how this has actually helped people. Maybe a bit of context about where we are coming from and where this might lead us... So its um, I think if things are fully explained, I think people will be quite happy to participate.’

Some of the gentlemen also highlighted the importance of explaining the research procedure because it enables the participant to realise that research is not always done for their personal benefit, but is instead conducted for the common good.

RESEARCH NURSE: *‘Do you all understand that by doing research it’s for the greater good, it’s not necessarily to improve your care or to change your treatments, it’s to help us understand who responds best to which types of treatment for the future?’*

PARTICIPANT: *‘That’s why you must provide the context. And, you know, give me this full understanding of, you know, we are getting this hormone treatment, we get it for a reason. Research has been undertaken which has lead us to this point.’*

### Word of mouth by other black prostate cancer sufferers

The concept of having a black prostate cancer sufferer available to promote participation in research to other black male prostate cancer sufferers was welcomed.

‘I think if you get people who have had treatment who have come out the other end to speak in a group like this for instance and explain that it is a good idea to have research because that research may well help your son, or your cousin.’

A number of individuals mentioned this idea in relation to research, explaining that it would be a good method of generating interest in studies.

There was also discussion of this theme in the context of getting black men to engage in their health in the first instance. There was mention of a mechanic who offered a free MOT to every black man who had received a prostate check at their GP surgery. When asked ‘do you think it helps the fact that he was a black man, who had been through it himself? And then he was then trying to engage people to come in?’ An individual responded, ‘yes, yes. That helped.’

### Advertisement

Many individuals suggested the use of leaflets and other media advertisements in order to promote research opportunities. Use of a culturally specific means of information dispersion may prove most effective, for example, newspapers specifically targeted at the black community. There also seemed to be differences in preferences within subgroups of the black community. For instance, it was noted that the African–French community would potentially show aversion to discussing health matters via word of mouth, and would instead prefer the use of television.

‘For the African-French community, they don’t like to pass information word from the mouth, because if you are sick, nobody wants to tell somebody else. In French community, they do not want to pass information onto somebody else. They keep secret. I’m sick, I can’t tell anybody. The only way to pass on that kind of information is by TV. We’ve got a lot of channels. Our community has got a channel, a television channel they watch everyday.’

### Incentives

Some individuals expressed positive opinions concerning the use of incentives to encourage individuals to participate in research, considering these a means of offsetting the financial and time constraints that may prevent an individual from participating in research.

‘Yes it would help, I think that things like that would help. It’s like an incentive really to, you know, leave your house or wherever you are. Or leave what you’re doing. You know to supplement you from, some people for instance they’re working they need to have something to supplement that, you know, because obviously we all have to live, right? I think that that could help.’

Interestingly, other individuals expressed concern and suspicion over the use of incentives.

‘The incentives, I’m a bit suspicious of incentives myself. Especially on something like health. The reason why I would want to participate in research is for the common good. If you put cash in front of me when you’re doing your research, I might suspect your motives! That is just me.’

## Findings in the context of the current literature

The literature review yielded 117 text results, some of which were utilised to gain an understanding of the current views held by the research community. Most of the above themes have also arisen in the literature surrounding the enrolment of black men into research projects. While some themes were more common than others, almost all were referenced to in at least two other studies. Table 5 provides an overview of the themes uncovered in this study as compared to some of the current literature. A key theme that has arisen in numerous other studies is the concept of mistrust, and the accompanying desire to have the research project thoroughly explained. Another key theme is that of financial and time constraints. While financial and time constraints were not explicitly mentioned in the focus group discussion, many of the reasons given for not participating in the focus group were related to the time constraints of their work.

Another theme uncovered was that of incentives and the mixed views which accompanied it. The literature predominantly presents the use of incentives favourably. However, the negative connotations of incentives, as expressed in this study, were much less common. It does not arise in any of the studies depicted in Table 5.

Another theme that has arisen in this study, but not in others, is the interest in herbal medicine. In contrast, our study population did not show concerns over confidentiality of the research studies [7, 11], lack of a culturally sensitive approach by researchers [4, 7, 8, 11, 13] and a fear of receiving the ineffective placebo drug and the side effects associated with trial medication [4, 5, 9, 10, 11, 13] – which have all been highlighted in the literature as potential barriers for recruitment.

## Discussion

Through conducting two focus groups with black prostate cancer patients, we identified that the predominant barrier to engagement in research was mistrust regarding researchers and participation in trials. To alleviate this mistrust, and to promote greater participation in research projects, participants strongly recommended that all research protocols are thoroughly explained, and that understanding is verified at numerous stages. Some individuals considered incentives valuable since they would protect against any loss of earnings from missing work or transport costs. However, others had the opposing view that incentives evoked suspicion.

Many of the themes that were uncovered from the focus groups are supported by current literature. A major theme within the literature is that of mistrust of the researchers and the concern that participants will be experimented on. This is likely to stem from historical instances as explained previously [5]. The general consensus for reducing this sense of mistrust is to provide extensive information about the study and to repeatedly highlight the importance of an individual’s welfare above the desire for data collection [4]. Essentially, participants do not want to feel as if any information is being withheld from them, and that they are aware of what to expect.

Another barrier to recruitment is a lack of understanding regarding what research entails and the origins of cancer [10]. Lack of this knowledge has been found to be a significant predictor of the willingness of African Americans to partake in research [14]. Our focus group was confused about the causes of cancer, which has already previously been implicated as a barrier to engagement in research [15]. A lack of understanding regarding research is often accompanied with a sense of fear of the research process, for instance concern about being prescribed the placebo drug, or the associated risks and side effects [16].

An additional barrier to recruitment that is discussed in the literature but was not explicitly uncovered in our study, is a lack of cultural sensitivity in the recruitment process [4]. This most likely reflects that our current practice is based on developing relationships with participants that are accepting and respectful of their cultural values and heritage [8]. Understanding the cultural beliefs of a community can help understand an individual’s aversion to participating in research. For instance, one cultural belief that became apparent in this study was the strong confidence in the efficacy of herbal medications. The influence of herbal medicine on participation is something which does not seem to have been considered in the literature so far. It is possible that strong beliefs in herbal medicine, which are commonly relied upon in the black community, could influence an individual’s decision to participate in research, especially as research tends to focus on pharmacologically-developed treatment and not natural remedies. By taking the time to explore these ideas, and helping the participants see their views in the context of research instead of as a separate, opposing entity, individuals may be more inclined to get involved.

Another cultural belief that has been documented in literature is a sense of fatality (i.e. the idea that life events are inevitable and out of an individual’s control) [7]. This may have links to belief in the power of God. As such, the use of the church as a recruitment strategy has been suggested in the literature [7].

Time and financial constraints are two other commonly reported barriers [10, 16]. As a means of resolving these issues, some form of compensation or incentive is readily recommended in the literature. The general consensus from a variety of studies is that incentives are viewed positively and may actively encourage research participation [11]. This view was also exemplified in this study. In some cases, financial incentives are even viewed as a requirement for participation. A survey by Shavers et al. of African–American residents found that the majority of individuals stated they would need payment for expenses (79%) and their time (64%) if they were to participate [9]. Interestingly, some individuals in this study had the opposing view that incentives evoke suspicion and would instead prevent participation in research. This is an idea that does not seem to be recognised in the literature. While further investigation into this newly identified belief would be required, it is clear that not everyone views money in the same way in the context of health. This requires careful consideration, as often studies appear happy to offer incentives with the assumption that this would improve recruitment, when in reality it could be having a previously unconsidered detrimental effect.

Two of the major facilitators for research recruitment, as identified in the literature, are the desire to have the research project thoroughly explained and (in some instances) the use of incentives. Another example, also referred to in our study, is the use of media advertisement (e.g. flyers, newspaper articles, television and radio). The use of particular channels, newspapers or radio stations that are favoured by the black community is likely to be most helpful. However, Swanson et al. warns against the use of mass media alone, instead suggesting that it should be utilised as a supplement to other recruitment strategies [13]. Other strategies discussed in the literature include establishing a site in the community for the study and providing alternative weekend and evening hours for the trial, in order to make the study as undisruptive as possible [13].

## Conclusion

Our focus groups were conducted in a way that enabled individuals to speak freely. There were set questions; however, participants were allowed to have discussions and to ask questions to one another and the focus group leaders. This encouraged individuals to speak about topics that perhaps they would have avoided had the focus group been more structured. However, there are also a number of limitations to the study. Firstly, the total number of participants is small. A greater number of focus groups would have enhanced the reliability of the opinions expressed, and may have gained different perspectives. Also, although the participants recruited were from mixed backgrounds, and therefore relatively representative, a specific strategy to ensure a truly representative sample was not utilised.

Ultimately, this study highlighted a number of important barriers and facilitators to recruitment into research, which may be useful in addressing the severe under-representation of black men in research studies. Although many of the themes identified in this study have been previously highlighted in the literature, the fact that these beliefs are still prevalent within the black community highlights a greater need to address them further. A number of novel concepts were uncovered, including the potential negative implications of financial incentives, and the implications of strong beliefs of herbal medication on the readiness to participate. It is also important to realise that the suggested strategies are only useful in combination, and that a blanket approach cannot be applied across the whole community due to the existence of unique subcultures and the differing preferences and beliefs that accompany them.

## Funding

This research was supported by the Experimental Cancer Medicine Centre at King’s College London and also by the National Institute for Health Research (NIHR) Biomedical Research Centre based at Guy’s and St Thomas’ NHS Foundation Trust and King’s College London. The views expressed are those of the author(s) and not necessarily those of the NHS, the NIHR or the Department of Health

## Figures and Tables

**Table 1.** Set questions asked in focus groups.

	Focus group questions
**1**	What is your understanding of medical research?
**2**	Has anyone ever approached you regarding taking part in research?
**3**	Have you ever seen any opportunities advertised or been aware of any research taking place?
**4**	Would you consider engaging in research opportunities?
**5**	What would prevent you from doing so?
**6**	What would encourage you?
**7**	Would you require incentives, for example, payment?

**Table 2.** Demographics of participants of both focus groups obtained via paper and telephone questionnaire.

	First focus group participant responses	Second focus group participant responses
**Mean age (SD)**	58 (10.7)	63 (3.8)
**Ethnicity (%)**		
Black–African	5 (50)	3 (50)
Black–Caribbean	3 (30)	3 (50)
White and Black–Caribbean	0 (0)	0 (0)
White and Black–African	0 (0)	0 (0)
Other	0 (0)	0 (0)
Missing	2 (20)	0 (0)
**Employment status (%)**		
Unemployed	0 (0)	1 (17)
Disabled	0 (0)	0 (0)
Retired	2 (20)	3 (50)
Part time	2 (20)	1 (17)
Full time	3 (30)	1 (17)
Sick leave	1 (10)	0 (0)
Missing	2 (20)	0 (0)
**Marital status (%)**		
Never married	1 (10)	1 (17)
Widowed	0 (0)	0 (0)
Divorced/separated	4 (40)	1 (17)
Married/long-term partner	3 (30)	3 (50)
Missing	2 (20)	1 (17)
**Distance from hospital (%)**		
Less than 5 miles	4 (40)	1 (17)
5–10 miles	4 (40)	1 (17)
10–15 miles	0 (0)	3 (50)
15 + miles	0 (0)	0 (0)
Missing	2 (20)	1 (17)

**Table 3.** Supporting quotes from focus group participants.

Theme	Quote
Mistrust	*‘But it's also to do with, people like us, you know, people of colour here, um, then there is a stigma also there. Thinking, you know, why are they trying to, you know, get us to do this and not go and ask somebody else. There's that type of feeling, you know, because there's a mistrust.’*
Lack of understanding	*‘We live, you see, I was born in Jamaica. Jamaica is in me. That's where my roots and everything are. As soon as you come out of that and enter into a different environment, the climate is different, the food is different, the air is different, the structure. So our body becomes changed.’*
Tendency to favour herbal medicine	*‘Well I'm looking at, because basically, we you see, in the West Indies, have lots of herbs and things like that what we boil when we have colds, when we're sick in our stomach and all those kind of things...Take Aloe Vera for one. Take pineapple for one. Take Chocho, Ladyfingers and Moringa and Guinea Hen weed. And all these things.’*

**Table 4.** Supporting quotes from focus group participants.

Theme	Quote
Explanations	*‘To me it’s just about explaining really. I think. I only speak on my experience, I don’t know what everybody thinks. But um, but I think if things are fully explained. If I understand the benefits of the research, including statistics about you know, how many people are affected, how this has actually helped people. Maybe a bit of context about where we are coming from and where this might lead us... So its um, I think if things are fully explained, I think people will be quite happy to participate.’* RESEARCH NURSE: *‘Do you all understand that by doing research it’s for the greater good, it’s not necessarily to improve your care or to change your treatments, it’s to help us understand who responds best to which types of treatment for the future?’* PARTICIPANT: *‘That’s why you must provide the context. And, you know, give me this full understanding of, you know, we are getting this hormone treatment, we get it for a reason. Research has been undertaken which has lead us to this point.’*
Word of mouth	*‘I think if you get people who have had treatment who have come out the other end to speak in a group like this for instance and explain that it is a good idea to have research because that research may well help your son, or your cousin.’*
Advertisement	*‘For the African-French community, they don’t like to pass information word from the mouth, because if you are sick, nobody wants to tell somebody else. In French community, they do not want to pass information onto somebody else. They keep secret. I’m sick, I can’t tell anybody. The only way to pass on that kind of information is by TV. We’ve got a lot of channels. Our community has got a channel, a television channel they watch everyday.’*
Positive incentive view	*‘Yes it would help, I think that things like that would help. It’s like an incentive really to, you know, leave your house or wherever you are. Or leave what you’re doing. You know to supplement you from, some people for instance they’re working they need to have something to supplement that, you know, because obviously we all have to live, right? I think that that could help.’*
Negative incentive view	*‘The incentives, I’m a bit suspicious of incentives myself. Especially on something like health. The reason why I would want to participate in research is for the common good. If you put cash in front of me when you’re doing your research, I might suspect your motives! That is just me.’*

**Table 5.** Themes identified versus current literature.

	Themes												
	Mistrust	Lack of understanding	Interests in herbal medicine	Concerns of confidentiality	Lack of cultural sensitivity	Financial and time constraints	Fear of placebo and side effects	Thorough explanations of research process	Word of mouth	Media advertisement	Positive views of incentives	Negative views of incentives	Use of the church
**Literature**													
Abernethy *et al* (2005)[7]													
Woods *et al* (2004)[8]													
Corbie-Smith *et al* (1999)[5]													
Shavers *et al* (2001)[9]													
Ford *et al* (2007)[10]													
George *et al* (2013)[11]													
Fouad *et al* (2001)[12]													
Swanson *et al* (1995)[13]													
Jones *et al* (2009)[4]													

**RED** = theme present in both this study and the literature.

**GREEN** = theme present in this study only.

**BLUE** = theme present in literature only.

**GREY** = theme absent from both this study and the literature.
